# Efficacy and safety of combined Xen Gel Stent-45 implantation and 25-gauge Pars Plana Vitrectomy: a case series

**DOI:** 10.22336/rjo.2023.58

**Published:** 2023

**Authors:** Federica Serino, Enrico Bernardi, Fabrizio Franco

**Affiliations:** * Eye Clinic, Department of Neurosciences, Florence, Italy; ** Department of Ophthalmology, Inselspital, University Hospital Bern, Switzerland

**Keywords:** MIGS, POAG, ERM, IOP

## Abstract

**Purpose:** To report outcomes in terms of efficacy and safety of patients affected with Primary Open Angle Glaucoma (POAG) and Vitreoretinal Disease, who have undergone Pars Plana Vitrectomy (PPV) and ab-interno XEN gel 45 (Abbvie) implantations.

**Methods:** This is a retrospective, observational, case series on five patients who underwent combined Pars Plana Vitrectomy and XEN gel Stent 45 implantation at “Careggi Hospital” Eye Clinic of Florence. Best-corrected visual acuity (BCVA) evaluation, intraocular pressure (IOP) measurements with Goldmann applanation tonometer (GAT), and several glaucoma medications were evaluated at the baseline and at one, three, six, and twelve months after surgery. Complications were recorded up to 1 year after surgery.

**Results:** 5 eyes in five patients were enrolled. IOP dropped from an average of 21,2 ± 3,3 mmHg preoperatively to 14,6 ± 1,1 mmHg at the end of the follow-up period (month 12), with a mean percentage reduction of 58%. One patient needed a needling procedure (20%). None needed reintervention. We did not register any case of hypotony (IOP < 6,5 mmHg), hypotony maculopathy and choroidal detachment. The postoperative number of anti-glaucomatous molecules was on average 0,2 ± 0,4.

**Conclusion:** Our results suggested that combined Pars Plana Vitrectomy and XEN gel stent 45 implantation is safe and effective for patients affected by visually significant vitreoretinal diseases and POAG.

**Abbreviations:** AC = anterior chamber, BCVA = Best-corrected visual acuity, ERM = epiretinal membrane, FTMH = full-thickness macular holes, FU = fluorouracil, GAT = Goldmann applanation tonometer, IOP = intraocular pressure, MIGS = minimally invasive glaucoma surgery, MMC = mitomycin C, NVG = neovascular glaucoma, OCT = optical coherence tomography, POAG = Primary Open Angle Glaucoma, PPV = Pars Plana Vitrectomy, SD = standard deviation, TB = Trabeculectomy, VF = visual field, VMI = Vitreomacular Interface, VMA = vitreomacular adhesion, VMT = vitreomacular traction

## Introduction

Vitreomacular Interface (VMI) disorders comprise a group of diseases such as vitreomacular adhesion (VMA), vitreomacular traction (VMT), epiretinal membrane (ERM), full-thickness macular holes (FTMH), and lamellar holes and pseudoholes. The progress of optical coherence tomography (OCT) technology has led to a dramatic improvement in visualizing and diagnosing VMI disorders, with a prevalence of up to 15.9% in individuals aged between 40 and 75 years [**1**]. Pars Plana Vitrectomy (PPV) is currently the mainstay of treatment for symptomatic VMD.

Meanwhile, Primary open-angle glaucoma (POAG) is a chronic, progressive, irreversible eye disease characterized by damage of the optic nerve and, if untreated, progressive visual field (VF) loss. Nowadays, it is a leading cause of irreversible preventable blindness worldwide [**2**]. 

Treatment in glaucoma management aims to lower intraocular pressure and, by doing so, to slow the rate of VF loss and disease progression [**3**]. IOP-lowering medications are usually recommended as the first-choice treatment [**4**]. Surgery is recommended whenever medical and laser treatments are ineffective to obtain an optimal reduction of IOP (target IOP). 

As technology in eye care progresses and the surgical time has reduced, the interest in combining multiple surgical procedures has increased. Potential advances to be considered are faster visual recovery and cost-effectiveness. 

Since POAG and VMI are both age-related conditions [**5**,**6**], they may coexist, and the surgeon may decide whether to perform both surgeries at the same time or treat them separately.

Trabeculectomy (TB) is generally an effective and safe procedure, and it is the most widely performed glaucoma surgical procedure; however, the procedure may lead to intraoperative (e.g. hemorrhage, choroidal effusion), postoperative (e.g. hypotony due to over filtration, hyphema, uveitis, endophthalmitis) or late-onset (e.g. leaking bleb, cataract) complications [**7**,**8**]. 

Postoperative hypotony is a common complication of trabeculectomy and tube implantation in vitrectomized eyes [**9**].

New minimally invasive glaucoma surgery (MIGS) devices have recently been developed to provide safer and less aggressive IOP lowering, as an alternative to surgical methods or laser procedures [**10**].

This case-series aim was to report the outcome in terms of efficacy and safety of patients affected with Primary Open Angle Glaucoma and Vitreoretinal Disease, who have undergone PPV and *ab-interno* XEN gel 45 (*Abbvie*) implantations.

## Materials and methods

This is a monocentric retrospective case series on 5 patients affected with POAG and ERM at the same time. All cases underwent contemporary PPV and Xen Gel 45 implantations. Three of the five cases involved a triple procedure. The study was conducted at the Eye Clinic, Neuromuscular and Sense Organs Department of Careggi University Hospital (Florence, Italy), and was performed according to the current version of the Declaration of Helsinki (52nd WMA General Assembly, Edinburgh, Scotland, UK, October 2000). All the patients included in the study signed a written informed consent.

All surgeries were performed by one of the authors (FF), between December 2021 and February 2022 at the Eye Clinic, Neuromuscular and Sense Organs Department of Careggi University Hospital (Florence, Italy). 

After disinfection of periocular skin and conjunctival fornix with povidone-iodine 10% and 5% respectively, sub-Tenon anesthesia with 4,5 ml of a mixture of ropivacaine, lidocaine, and Hyaluronidase was done. If a combined surgery was necessary, phacoemulsification was performed at the beginning of the procedure, implanting monofocal intraocular lenses (IOLs). Thereafter, three port 25-gauge PPV and membrane peeling with the use of a vital dye (Brilliant Blue) were performed in the standard fashion (Constellation, Alcon): the trocars were inserted in the pars plana (3.0-4.0 mm from the limbus depending on the lens status) in the superior-temporal, inferior-temporal and superior-nasal quadrants. A suture was placed for any wounds that were not self-sealing.

**Fig. 1:**
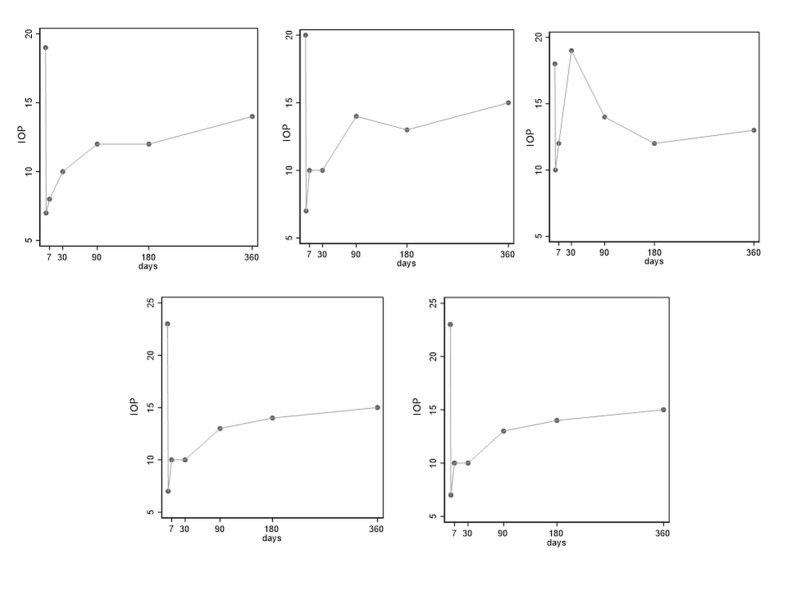
Graphs showing the changes in intraocular pressure (IOP) for each patient (IOP = intraocular pressure)

Finally, we proceed to the XEN Gel Stent Implantation through the “Sparkling XEN Technique” as previously described by the authors [**11**]. The area of the future bleb was identified in the inferior nasal quadrant since it was spared from inserting trocars. The first step was the dissection of the subconjunctival space: we entered the conjunctiva at 3 mm from the limbus with a 27-gauge needle on an insulin syringe and positioned the bevel up very superficially beneath the conjunctiva avoiding perforating the Tenon’s capsule; then we arrived in the marked target area directing the tip of the needle posteriorly. At this point, 0,1 mL of air was injected; if the pneumo-dissection was successful, bubbles would form in subconjunctival space (**Fig. 1**). Then, 0,1 mL of BSS was injected. Once the conjunctiva was well separated from Tenon’s capsule, we implanted the Xen Gel Stent with the traditional ab-interno approach. A cohesive viscoelastic (Healon GV Pro, Johnson & Johnson) was injected to fill the anterior chamber (AC) through a clear corneal incision. The disposable injector entered the AC through a main corneal incision in the inferotemporal sector, upon staining the stent with a blue dye (Trypan blue), and was directed toward the superonasal angle. An indirect gonio lens was used to watch the angle (a direct view gonio lens can also be used): the correct placement was just anterior to the pigmented trabecular meshwork. Once the needle tip was in the correct place, it was pushed forward through the sclera coming out in the subconjunctival space 3.0 mm away from the limbus. The injector was actioned, and the needle retracted into the sleeve. The device was correctly positioned at 1 mm in the AC, 2 mm in the scleral tunnel, and 3 mm in the subconjunctival space. The viscoelastic was removed from the anterior chamber with balanced salt solutions. Finally, we performed a subconjunctival injection of 0,1 mL of mitomycin C (MMC) 0,02% in the bleb.

Patients were instructed to discontinue all glaucoma medications on the day of the surgery; follow-up visits were conducted on the postoperative day 1, every week for the first month, at month 3, month 6, and month 12, and included anterior segment biomicroscopy, fundus oculi examination and IOP measurements (GAT). Assessments of bleb morphology using Anterior Segment Optical Coherence Tomography images (AS-OCT, MS39 CSO Scandicci) and SD-OCT assessment of the macula (Heidelberg Spectralis) were performed at postoperative visits on day 7 and month 1, and whenever required. Post-operative therapy included antibiotic prophylaxis for 1 week and steroids tapered in 3 months.

## Results

We reported our data on 5 eyes of 5 patients all with a diagnosis of POAG. Patients were also affected with idiopathic epiretinal membrane, with no previous history of intraocular surgery except for uncomplicated phacoemulsification. Two eyes (40%) were already pseudophakic, and 3 eyes were phakic and underwent simultaneous cataract surgery, PPV, and XEN implantation. **Table 1** summarizes the patients’ characteristics herein described. The preoperative number of anti-glaucomatous molecules was on average 2,2 ± 0,4.

**Table 1:** Baseline patients’ characteristics

Variable	Overall (n=5)
Age (mean value ± SD)	67 ± 9
Sex (Male/Female)	3/2
Type of glaucoma:	
POAG	5 (100%)
ERM Stages:	
- Stage 2	1 (20%)
- Stage 3	4 (80%)
Previous surgery:	
- Phaco + IOL	2 (40%)
Number of antiglaucoma medications	2,2 ± 0,4
SD = standard deviation, POAG = primary open angle glaucoma, Phaco + IOL = Phacoemulsification + intraocular lens implantation	

We obtained a good reduction of the IOP from an average of 21,2 ± 3,3 mmHg preoperatively to 9,8 ± 1,5 mmHg 7 days after the surgery. This result persisted through the follow-up: at 1 month, the average IOP was 12,2 ± 3,9 mmHg, at 3 months it was 13,4 ± 0,9 mmHg, at 6 months 13,4 ± 1,7 mmHg, and 12 months 14,6 ± 1,1 mmHg (**Table 2**). 

**Table 2:** IOP reduction from baseline

Patients	IOP (mmHg) baseline	IOP (mmHg) day 1	IOP (mmHg) day 7	IOP (mmHg) month 1	IOP (mmHg) month 3	IOP (mmHg) month 6	IOP (mmHg) month 12
1	19	7	8	10	12	12	14
2	20	7	10	10	14	13	15
3	18	10	12	19	14	12	13
4	23	7	10	10	13	14	15
5	26	8	9	12	14	16	16
Mean ± SD	21,2 ± 3,3	7,8 ± 1,3	9,8 ± 1,5	12,2 ± 3,9	13,4 ± 0,9	13,4 ± 1,7	14,6 ± 1,1
IOP = intraocular pressure, SD = standard deviation							

The probability of complete success meant that IOP ≤ 18 mmHg at month 12 without the need for surgically revising the bleb or reoperation, was 100%. Only one patient needed a needling procedure (patient 3) (20%). No patient needed reintervention because of uncontrolled IOP. 

We did not register any case of hypotony (IOP < 6,5 mmHg), hypotony maculopathy and choroidal detachment. 

The mean BCVA at baseline was 0,52 ± 0,1. At month 12, the mean BCVA was 0,24 ± 0,1 logMAR. 

During the follow up we reintroduced one anti-glaucomatous molecule in one patient (20%). The postoperative number of anti-glaucomatous molecules was on average 0,2 ± 0,4. 

## Discussion

The effectiveness and safety of combined surgery for co-existing cataract and glaucoma have been widely investigated, with non-reporting statistically significant differences concerning intraoperative or postoperative complications [**12**]. The outcomes of combined PPV and Filtering Surgery have been less investigated, instead. 

However, the prevalence of VMI disorders, exactly like POAG, increased with aging. Sometimes, these can lead to serious conditions associated with visual disturbances, such as metamorphopsia and diminished visual acuity, requiring surgical intervention. An association between these disorders has been hypothesized: the increased oxidative stress in patients with glaucoma has been linked to vitreous changes [**13**]. But, a previous study did not find any statistically significant association between glaucoma and VMI disorders [**14**].

This retrospective case series aim was to report our experience with combined PPV and XEN 45 Gel Stent Implantation and our outcomes in terms of efficacy and safety.

Regarding the latter, hypotony is a significant complication that has been associated with delayed visual recovery following filtering surgery, especially following the use of adjuvant antimetabolites such as MMC or 5 fluorouracil (5FU) to prevent postoperative fibrosis [**15**]. While statistical hypotony is defined as an IOP, being less than 6.5 mmHg (more than 3 standard deviations below the mean of IOP of a general non-glaucomatous population) [**16**], clinically significant hypotony can be defined as the condition in which the IOP is low enough to result in visual loss. Hypotony has been related to the intraoperative use of MMC, as well as various color dyes used for epiretinal membrane surgery [**17**]. These medications may have cytotoxic effects on the ciliary body, leading to decreased aqueous production.

If early post-operative hypotony occurs in a non-vitrectomized eye, when the aqueous component is completely depleted, the vitreous remains in the eye, preventing the IOP from going to zero. In contrast, in a vitrectomized eye, a continuous leakage can abolish the IOP, and a choroidal detachment could occur, in the absence of an inner opposing force and with a lower scleral rigidity [**18**].

XEN Gel Stent has been designed to limit hypotony, and lower IOP safely in a predictable manner by applying the Hagen-Poiseuille equation. This law postulates that the pressure differential across a tube with constant dimensions is proportional to the resistance to flow, and this is directly proportional to the length but inversely proportional to the radius of the tube to the fourth power. A tube with a length of 6 mm and 45 μm of lumen size of diameter at average aqueous humor production of 2–3 μl/min provides a theoretical pressure drop of 8 mmHg, which reduces the possibility of post-operative hypotony [**19**]. There are indeed no cases of hypotony, hypotony maculopathy, or choroidal detachment. This is interesting whether we compare the results of other filtration procedures in vitrectomized eyes: Erçalik et al. reported a rate of 38,4% of hypotony with Ahmed Valve Implantation [**20**]; Hong et al. reported a rate of 11.8% again with Ahmed Valve Implantation [**21**]; Van Aken et al. reported a rate of 17% of hypotony and 3% of suprachoroidal hemorrhage with Baerveldt Glaucoma Implants [**22**]. In addition, the probability of success was lower in these previous studies if compared with our results. However, we believe that our data cannot be compared with the above results because these examined Secondary Glaucoma following pars plana vitrectomy. To our knowledge, this is the first report about combining PPV with a XEN Gel Stent to treat patients with POAG. 

Regarding the efficacy issue, it is well known that any vitreoretinal procedures make glaucoma surgery challenging. Inserting the trocars causes scarring of the conjunctiva, increasing the risk for bleb scarring and, thus, surgical failure [**23**]. Moreover, PPV causes an increase in oxygen levels in the vitreous chamber, thus increasing oxidative damage to the trabecular meshwork [**24**]. Therefore, a previous study reported that trabeculectomy after PPV has a lower success rate than without combined PPV in eyes with neovascular glaucoma (NVG) [**25**,**26**]. Our study considered different baseline pathologies (POAG and ERM), and thus it is difficult to compare our results with the findings of these previously cited studies. Anyway, despite the presence of the risk factors cited, the probability of complete success was total (100%) in our case series. The mean pre-operative IOP was 21,2 ± 3,3 mmHg. At month 12, IOP was 14,6 ± 1,1 mmHg with a reduction of 58% as compared to the preoperative value. At month 1, patient 3 had an IOP spike: thus, we performed a needling procedure which was unsuccessful. We decided to reintroduce a single anti-glaucomatous molecule, which controlled the IOP at the end of the follow-up period. The needling rate was 20%, which is consistent with what is found in the literature [**27**]. The number of IOP-lowering medications meant as the number of active principles, was on average 2,2 ± 0,4 and dropped to 0,2 ± 0,4 at month 12 (percentage decrease of 45%). Toussaint et al. reported their experience with another MIGS, the Trabectome, combined with PPV, to treat POAG, with similar results [**28**]. 

## Conclusion

We reported our good results on the efficacy and safety of combined PPV and XEN Gel Stent Implantation in patients affected with POAG and Vitreoretinal Interface Disorders (ERM). We believe that, with improvements in technologies in recent years and the attention on the cost issue, the possibility of combining different surgical procedures has become even more important, allowing to reduce surgical and visual recovery time. Our results showed that by combining the procedures we can obtain excellent visual and IOP results, without an increase of complications. The limits of this study were certainly the retrospective design and the small sample size, but our work could be a starting point for an interesting surgical issue that is worth investigating. 

**Conflict of Interest Statement**

The authors have no conflicts of interest to declare.

**Informed Consent and Human and Animal Rights Statement**

Informed consent was obtained from all subjects involved in the study.

**Authorization for the use of human subjects**

Ethical approval: The research related to human use complied with all relevant national regulations and institutional policies, followed the tenets of the Helsinki Declaration, and was approved by the Institutional Review Board of Careggi University Hospital, Florence, Italy. 

**Acknowledgments**

None.

**Sources of Funding**

This research received no external funding.

**Disclosures**

None.
